# Influence of Rapid Freeze-Thaw Cycling on the Mechanical Properties of Sustainable Strain-Hardening Cement Composite (2SHCC)

**DOI:** 10.3390/ma7021422

**Published:** 2014-02-24

**Authors:** Seok-Joon Jang, Keitetsu Rokugo, Wan-Shin Park, Hyun-Do Yun

**Affiliations:** 1 Department of Architectural Engineering, Chungnam National University, Daejeon 305-764, Korea; E-Mail: suk-joon@nate.com; 2 Department of Civil Engineering, Gifu University, Gifu 501-1193, Japan; E-Mail: rk@gifu-u.ac.jp; 3 Department of Construction Engineering Education, Chungnam National University, Daejeon 305-764, Korea; E-Mail: salshin@cnu.ac.kr

**Keywords:** strain-hardening cement composite (SHCC), freeze-thaw, sustainable material, compressive behavior, flexural behavior, direct tensile behavior, splitting tensile strength

## Abstract

This paper provides experimental results to investigate the mechanical properties of sustainable strain-hardening cement composite (2SHCC) for infrastructures after freeze-thaw actions. To improve the sustainability of SHCC materials in this study, high energy-consumptive components—silica sand, cement, and polyvinyl alcohol (PVA) fibers—in the conventional SHCC materials are partially replaced with recycled materials such as recycled sand, fly ash, and polyethylene terephthalate (PET) fibers, respectively. To investigate the mechanical properties of green SHCC that contains recycled materials, the cement, PVA fiber and silica sand were replaced with 10% fly ash, 25% PET fiber, and 10% recycled aggregate based on preliminary experimental results for the development of 2SHCC material, respectively. The dynamic modulus of elasticity and weight for 2SHCC material were measured at every 30 cycles of freeze-thaw. The effects of freeze-thaw cycles on the mechanical properties of sustainable SHCC are evaluated by conducting compressive tests, four-point flexural tests, direct tensile tests and prism splitting tests after 90, 180, and 300 cycles of rapid freeze-thaw. Freeze-thaw testing was conducted according to ASTM C 666 Procedure A. Test results show that after 300 cycles of freezing and thawing actions, the dynamic modulus of elasticity and mass loss of damaged 2SHCC were similar to those of virgin 2SHCC, while the freeze-thaw cycles influence mechanical properties of the 2SHCC material except for compressive behavior.

## 1. Introduction

Cement material, such as concrete, is one of the most widely used structural materials for civil infrastructures. However, this material is typically characterized as brittle, with a low tensile strength and strain capacity. During the past five decades, many researchers have tried to overcome this weakness of cement composite. It is known that adding discontinuous fibers to cement material, resulting in fiber-reinforced cement composites (FRCCs), improves tensile performance as well as many engineering properties of cement composite due to the fibers bridging the cracks through bond between fibers and cement matrix [1].

Conventional FRCCs are characterized by quasi-brittle, or strain softening, behaviors in tension, with first cracking followed by immediate crack localization [2,3]. This behavior limits the FRCC primarily to non-structural applications. The FRCCs are manufactured by simply adding short fibers to the cement matrix in relatively low volume fractions. In recent years, a new generation of FRCC, which exhibits multiple cracking and tensile strain-hardening behaviors after matrix first cracking under direct tension, offer ideal and low-cost solutions for repair, retrofitting, and new construction of civil infrastructure [4]. This type of FRCC is referred as strain-hardening cement composite (SHCC) and is a FRCC that is micro-mechanically tailored by the addition of short fibers to obtain high ductile and multiple cracking behaviors under direct tension. To achieve strain-hardening and multiple cracking behaviors in the FRCC, only a little amount of silica sand and higher water-to-binder ratio (W/B) are allowed to be used in the cement composite in order to control fracture toughness of cement composite. The cement composite should also contain moderate volume fraction between 1% and 2% of synthetic fibers to bridge the cracks and redistribute stress in the cement composite. Coarse aggregates are eliminated in the cement composite also, resulting in higher cement content compared with conventional concrete [5].

Reinforced concrete is the most popular construction material for civil infrastructures, which provide essential services to our society and economy. The production of cement, a key component of concrete mix, emits 5% of global greenhouse gas emission created by human activities [6]. Thus, it is logical to incorporate life cycle and sustainability concerns into the design of civil engineering materials. It is not except for high performance FRCCs such as SHCC materials. Choi *et al.* [7] showed that 2SHCC material used in this study reduced carbon dioxide (CO_2_) emissions by about 20%, although this estimate can be vary depending on local conditions at the source of raw materials, binder quantity and amount of Ordinary Portland cement (OPC) replacement, type of manufacturing facilities, climate, energy sources, and transportation distances.

Li *et al.* [8] reported that the concept of green engineered cementitious composite (ECC) for sustainable infrastructures is feasible based on the some preliminary experimental results of the effect of about 70% cement substitution with industrial by products, such as two types of fly ash and one type of bottom ash, on the mechanical properties. Lepech *et al.* [9] proposed the green ECC utilizing low cement content and replacing virgin sand with waste foundry sand. Yang *et al.* [10] proposed the green ECC that replaced maximum 85% of cement by weight to fly ash. Qian *et al.* [11] developed the sustainable SHCC incorporating blast furnace slag (BFS) and limestone powder and also evaluated the self-healing behavior of the pre-cracked SHCC specimens. Zhou *et al.* [12] used limestone power and BFS to produce green ECC. Their test results indicated that the increasing limestone power and BFS contents lead to a smaller average loaded crack width. Kim *et al.* [13,14] investigated the effect of recycled material in the SHCC on the mechanical properties to promote SHCC sustainability in infrastructure design through the use of recycled materials. Alternative recycled materials—recycled sand from demolished concrete, fly ash, and polyethylene terephthalate (PET) fibers—are used to partially replace silica sand, cement and polyvinyl alcohol (PVA) fibers, respectively, in SHCC specimens. Choi *et al.* [7] proposed that based on the performance requirements for SHCC, the allowable replacement rate is applicable up to 20% for fly ash, 50% for recycled aggregate and 20% for PET fiber along with fly ash replacement. For reducing greenhouse gases emission in the ECC containing high cement content, palm oil fuel ash (POFA) was used for partial cement replacement in the ECC [15]. The flexural performance of green ECC including high volume of POFA was investigated for three sets of ECC with water-to-binder-ratios (W/B) of 0.33, 0.36, and 0.38. Test results suggested that higher POFA content for each mixture improved the flexural deflection capacity and cracking behavior of green ECC prism specimens. Huang *et al.* [16] provided the results of an initial attempt of utilizing iron ore tailings (IOTs) to develop greener ECC. From their study results, it is reported that the replacement of cement with IOTs results in 10%–32% reduction in energy consumption and 29%–63% reduction in carbon dioxide emissions in green ECC compared with typical ECC. Aldahdooh *et al.* [17] investigate the potential of green ultra-high performance fiber reinforced cementitious composites in which 75% cement binder was replaced with ultrafine POFA. It is noted that ultrafine POFA possesses significant potential as an efficient pozzolanic mineral admixture for the production of green ultra-high performance fiber reinforced cementitious composite (UHPFRCC) with possibly superior engineering properties. Yun *et al.* [18] investigated the frost resistance and direct tensile properties of SHCC incorporating recycled aggregate, recycled fibers and fly ash after rapid freezing and thawing exposure.

Existing studies described above have focused on the substitution potential of partial portion of the cement in ECC with industrial wastes or by-products as supplementary cement-based material without sacrificing its mechanical properties, particularly its ductility and cracking behavior. From these studies, the feasibility of partially replacing the ECC’s components, such as cement, fiber and silica sand, with industrial by-products or recycled materials has been noted to reduce negative environmental impacts due to carbon dioxide emission.

Freeze-thaw cycles can be harmful to porous and brittle cement composite such as mortar and concrete. Therefore, freezing-thawing durability of cement composite has a significant effect on the service life and quality of infrastructures constructed with cement composite, especially exposed to cold weather environment. Resistance of concrete to freezing and thawing has been reported on the many available literatures [19,20]. The use of air-entraining agents and short fiber reinforcement results in the high resistance of concrete to severe frost action, or cycles of freezing and thawing [21–24]. Conventional HPFRCCs including SHCC and ECC have a good frost resistance because they have proper air-void system and short fibers [25–28]. It is known that the resistance of cement composite to freeze-thaw cycles depends on mixture component and composition. Although mechanical properties of sustainable SHCC including recycled or by-product materials have been investigated as described above, little quantitative data has been published in the scientific and technical literature on the freeze-thaw durability of sustainable SHCC material. Freeze-thaw durability properties of 2SHCC material used for civil infrastructures in cold regions must be evaluated. This study pioneers research on the resistance to freeze-thaw action in 2SHCC, an area unexplored to date.

In previous work [7,13,14], the authors proposed the allowable replacement rate of recycled components for 2SHCC material based on the mechanical properties of 2SHCC materials with different replacement levels of recycled components. This study was conducted to comprehensively examine the frost resistance and mechanical performances of 2SHCC material subjected to freezing and thawing cycles. The resulting data is valuable in view of the growing utilization of 2SHCC for durable infrastructures and the reduction in carbon dioxide emissions of concrete industry.

## 2. Experimental Investigation

### 2.1. Materials, Mix Proportion and Mixing

Based on previous studies [7,13,14], a sustainable SHCC mixture containing recycled sand, PET fibers, and fly ash was designed. Table 1 gives the mix proportion of the 2SHCC mixture in which raw materials were partially replaced with recycled material; recycled sand (a maximum size of 3.0 mm) from demolished concrete wastes, Class F fly ash and PET fibers from recycled bottles. The cement used in this study is Ordinary Portland cement (OPC). The specific gravity and water absorption for recycled sand were 2.49 g/cm^3^ and 3.0%, respectively. The fine silica sand with specific gravity of 2.61 g/cm^3^ and an average size of 110 μm were used. PVA fibers (REC 15) from Japan were used in this study. PVA fibers with a diameter of 40 μm and length of 12 mm were purposely manufactured with a tensile strength of 1600 MPa and elastic modulus of 40 GPa. The length and diameter of the PET fibers were 10 mm and 33 μm, respectively. The chemical additives used were a dry viscosity agent (methyl cellulose, MC) and polynaphthalene sulfonate-based superplasticizer (SP). A small amount of antifoaming agent was also added to control the microvoids generated by the MC.

To investigate the mechanical properties of 2SHCC material that contains recycled materials, the cement, PVA fiber and silica sand were replaced with 10% fly ash, 25% PET fiber, and 10% recycled sand based on preliminary experimental results [7,13,14] for the development of recycled SHCC, respectively.

Dry components in 2SHCC were first mixed with pan mixer until a homogeneous mix was reached at low speed. Mixing water and superplasticizer were added and mixed at low speed for 1 min and then at high speed for 2 min. After synthetic PVA and PET fibers were spread on the mixed cement mortar and mixed at high speed for 2 min. The fresh 2SHCC was cast into 100 mm × 200 mm cylindrical, 100 mm × 100 mm × 450 mm prismatic and dumbbell-shaped specimen molds. All specimens were removed from the molds 24 h after casting and then cured in a standard curing room (20 ± 3 °C and 95% RH) for another 13 days.

Geissert *et al.* [29] proposed a composite prism splitting test specifically for evaluating the bonding durability of two different cement composites affected by freeze-thaw cycling. In present study, the bond strength of 2SHCC exposed to freezing and thawing action was evaluated by a composite prism split testing method. For evaluating bond strength between substrate concrete and 2SHCC material, substrate concrete with 50 mm thickness was cast in prismatic molds and the molds were cured in standard curing room for 24 h. All bond surfaces of the substrate concrete were lightly roughened using a steel wire brush to remove slurry cement from external surface of both fine and coarse aggregates. Then 2SHCC material with 50 mm thickness was cast. The composite prisms for split tests were cured 24 h more than other specimens at standard curing room and then demolded.

A part of specimens was placed in the freeze-thaw chamber after a curing age of 14 days. The rest of the specimens were kept as reference specimens in the standard curing room.

### 2.2. Testing Methods

A freezing and thawing testing apparatus satisfying the ASTM C666 Procedure A (rapid freezing and thawing in water) requirements was used for testing. The freeze–thaw cycle consisted of alternatively lowering the temperature of the specimens from 4 to −18 °C and raising it from −18 to 4 °C in 4.0 h.

The mechanical properties of virgin 2SHCC materials under compression, flexure, direct tension and split testing for bonding strength were obtained by testing three companion specimens at the curing age of 28 and 74 days corresponding to 90 and 300 cycles of rapid freezing and thawing. These experimental results were used for comparison with the mechanical properties of SHCC materials exposed to 90, 180, and 300 cycles of rapid freezing and thawing.

The compressive strength test, conducted on the three cylindrical specimens at predetermined cycles of freezing and thawing, followed ASTM C39 test for compressive strength of cylindrical concrete specimens. All of the compressive tests were performed at a constant rate of 0.5 MPa/s until maximum strength and then controlled by displacement up to failure.

The flexural strength test, conducted using prismatic specimens under four-point flexural loading, followed the ASTM C1018 test method. All flexural specimens were loaded with 200 kN capacity MTS testing machine under displacement control with a loading rate of 0.20 mm/min.

**Figure 1.**
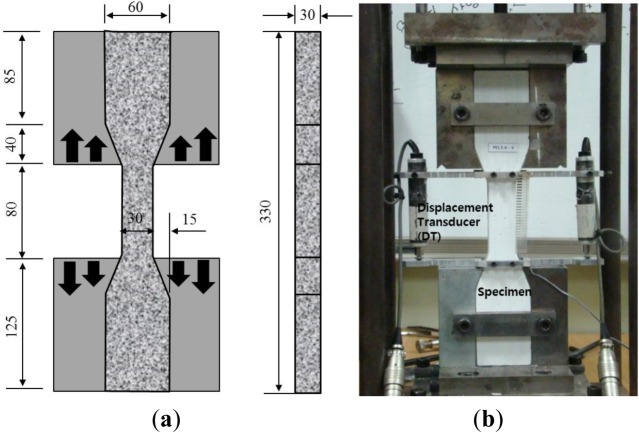
Test specimen and apparatus for direct tension. (**a**) geometry of tensile specimen (unit: mm); (**b**) test set-up.

The JSCE’s recommendations [30] were used to measure the direct tensile strength of dumbbell-shaped specimens. The geometry of tensile specimen and direct tensile loading apparatus used are depicted in Figure 1. The section of tensile specimens is 30 mm × 30 mm, and the gage length of the specimen is 80 mm.

For the tensile loading, two identical loading fixtures were used: one hinge chuck that is connected to the loading bar from a hand-cranked loading jack placed on the steel frame; the other fixed chuck is mounted on the bottom base of the steel frame. The upper fixture is pulled by the load along the two guide pins. Tensile loads were introduced to the tensile specimens along the central axis while the upper fixture was being pulled. The applied tensile loads were measured using a load cell with a capacity of 100 kN installed in the upper part of the loading device. Two displacement transducers (DTs) were mounted on the two sides of the specimen and used for taking deformation measurements as well as for test control. The displacement of the center 80 mm region of the dumbbell-shaped specimen was measured by means of two DTs, and the tensile strain was calculated by dividing this measured displacement by the reference length of 80 mm. DT holders were designed specially to allow the DT transducers to be easily adjusted to accommodate centering and offsetting. All the specimens were tested up to complete tensile failure. All of uniaxial tensile tests were conducted at a constant engineering strain rate of 0.5 percent/min.

**Figure 2.**
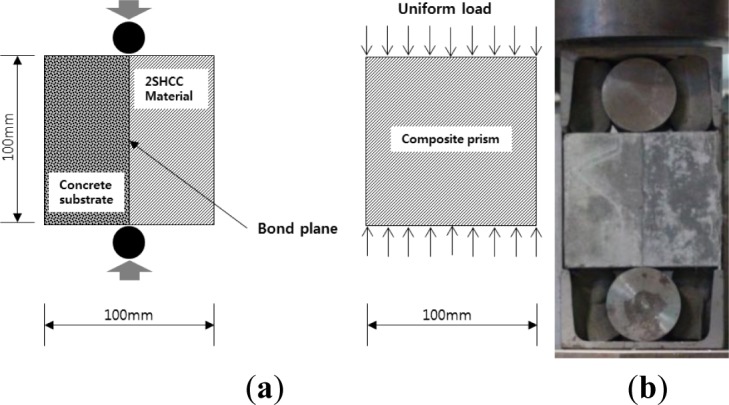
Splitting test specimen and apparatus for bond strength. (**a**) splitting tensile specimen; (**b**) test set-up.

To determine the bond strength between substrate concrete and 2SHCC material after exposure to freezing and thawing action, first the composite prism with dimension of 100 mm × 100 mm × 450 mm, to fit the freeze-thaw apparatus, was made. Prior to splitting test, a composite prism subjected to freeze-thaw cycling is saw-cut into four smaller cubic blocks with the size of 100 mm × 100 mm × 100 mm. About 25 mm at each end of composite prism is discarded. The test cubic block is placed in 200 kN universal testing machine (UTM) so that the interface between substrate concrete and 2SHCC material is vertical and the two opposing compressive line loadings are applied along the sawed interface. The test specimen and set-up are shown in Figure 2. As applied in the reference [28], vertical load was supplied at the rate of 8 kN/min.

## 3. Results and Discussions

### 3.1. Resistance to Freezing and Thawing

To evaluate the frost resistance of 2SHCC, the relative dynamic modulus and weight change over the freezing and thawing cycles were measured and recorded. The variation in the both values over the entire duration of freezing and thawing cycles provides a good indication of the deterioration of cement composites.

**Figure 3.**
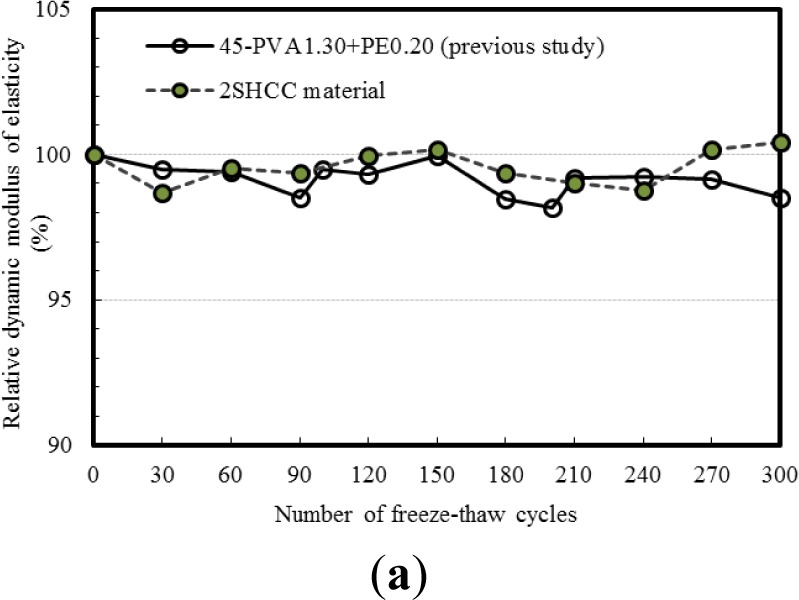

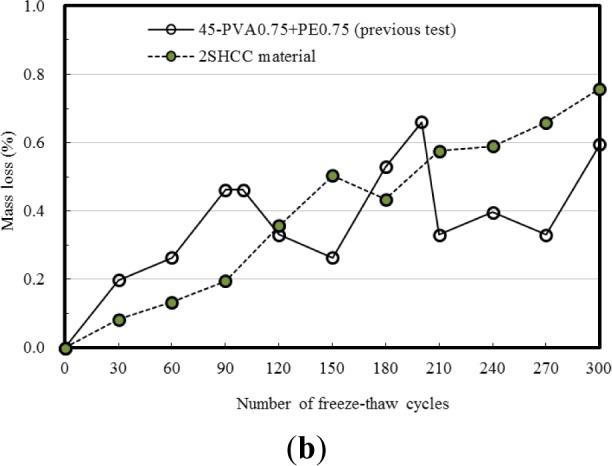
Freeze-thaw resistance of 2SHCC material. (**a**) relative dynamic modulus of elasticity; (**b**) mass loss.

Figure 3a,b provide the variation in relative dynamic modulus and mass loss of 2SHCC prism specimen with respect to the number of freeze-thaw cycles, respectively. Figure 3 includes the resistance to freezing and thawing of SHCC material [28] mixed with raw components for comparison with that of 2SHCC material replaced with recycled components. As shown in Figure 3, both strain-hardening cement composites (SHCCs) with raw or recycled components survived more than 300 freezing and thawing cycles. This significant improvement was due to an adequate size range and number of air void entrained by fly ash, silica fume and synthetic fibers in sustainable and conventional SHCCs [25,28,31,32]. Resistance to freezing and thawing of 2SHCC proposed in the study shows equal durability characteristics compared to that of the conventional SHCC material up to 300 freeze-thaw cycles.

### 3.2. Compressive Behavior

Compression test results of cement composite such as concrete and mortar represented the mechanical characterization of the material and the evaluation of its compressive strength. The data on the compressive strength are summarized in Table 2. For the various curing ages and cycle number of freezing and thawing, the average compressive strength of three cylinders varied from 35.4 to 46.3 MPa. As can be seen from Table 2, the compressive strength was little affected by the freezing and thawing action within 300 cycles. For lightweight concrete, high performance concrete, fiber reinforced concrete and strain-hardening cement composite (SHCC), there were not found significant differences between virgin and freeze-thaw attacked cylindrical specimens in the value of the compressive strength and static modulus of elasticity [28,33–35]. The results of these studies on effect of freeze-thaw actions on the compressive behavior of various cement composites using virgin components are similar to the test results of this study on the sustainable SHCC including recycled components.

**Figure 4.**
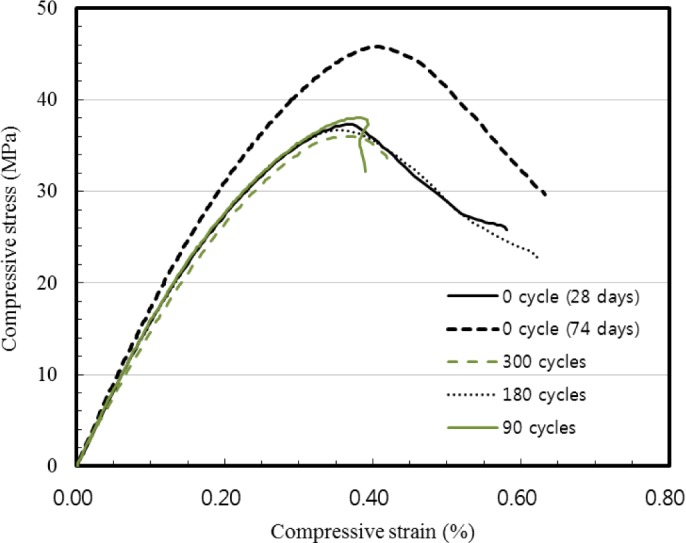
Typical compressive stress-strain curves of 2SHCC cylinders.

Complete stress *versus* strain curves of 2SHCC were obtained from the compression tests of the cylinders with a controlled displacement. Typical compressive stress *versus* strain curves of 2SHCC cylindrical specimens at a curing age of 28 and 74 days and after 90, 180 and 300 cycles of freezing and thawing are showed in Figure 4. Three cylinders were tested at each curing age and freeze-thaw cycle. Among the test results, a typical curve for each condition was provided in Figure 4. The sustainable SHCC material remains virgin in terms of overall compressive behavior after freezing and thawing exposure.

Measured compressive stress and strain curves indicated that compressive behavior of 2SHCC material is little affected by freezing and thawing actions. However, compressive behavior of 2SHCC at a curing age of 74 days depends on the exposed environment such as exposure to freeze-thaw cycles or standard curing. For 2SHCC cylindrical specimens with the same curing age of 74 days and different curing condition, average compressive strength of 2SHCC cylinders kept at the standard curing room is about 25% higher than that of 2SHCC specimens exposed to freeze-thaw environment. Likely reason for the lower compressive strength of freeze-thaw damaged 2SHCC cylinders is the maturity difference between cylindrical specimens exposed to freeze-thaw conditions and cylinders stored in a standard curing room [35].

**Figure 5.**
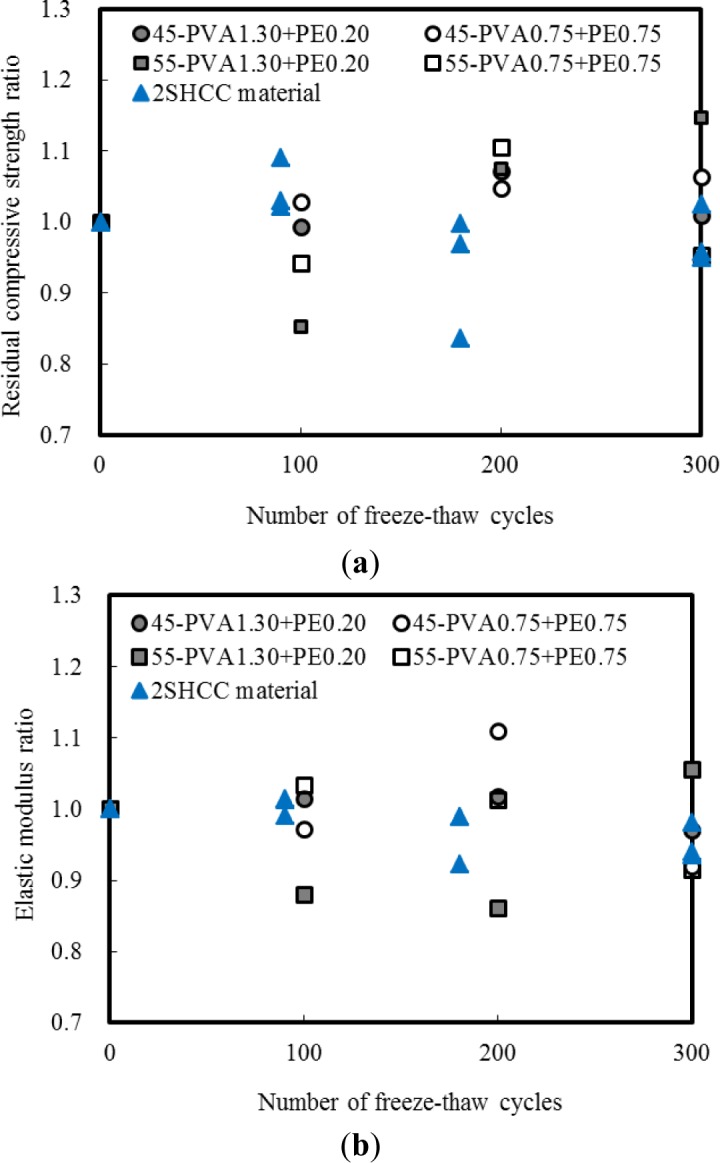
Compressive characteristics of sustainable and conventional SHCC cylinder. (**a**) compressive strength ratio; (**b**) static modulus of elasticity ratio.

Figure 5 shows the variations in compressive strength ratio and the static modulus of elasticity ratio with respect to the number of freeze-thaw cycles for sustainable and conventional SHCC cited from author’s previous study [28]. The compressive strength ratio is defined as the compressive strength of sustainable and conventional SHCC at a given freeze-thaw cycle divided by the compressive strength of SHCC materials without exposure to freeze-thaw cycle. The elastic modulus ratio is also defined by the same concept. Freeze-thaw actions up to 300 cycles lead to about 10% difference in the compressive strength and elastic modulus of sustainable and conventional SHCC materials before and after freezing and thawing cycles.

### 3.3. Flexural Behavior

**Figure 6.**
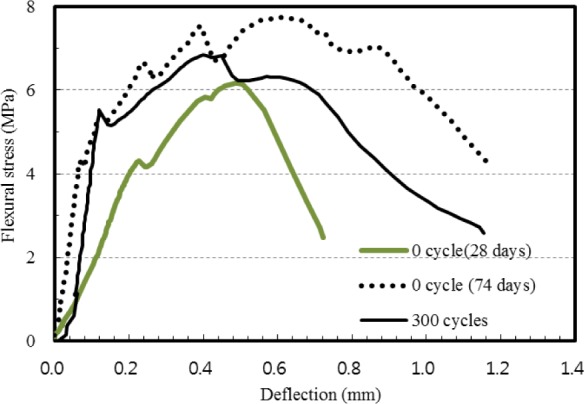
Typical flexural responses of 2SHCC prisms.

Figure 6 shows typical flexural stress *versus* deflection curves of 2SHCC prismatic specimens at a curing age of 28 and 74 days, and after 300 cycles of freezing and thawing. In Figure 6, the maximum flexural stress is defined as the flexural strength, and the corresponding deflection is defined as the flexural deflection capacity. From the comparison of the flexural responses of the 2SHCC prismatic specimens shown in Figure 6, it can be seen that freezing and thawing exposure makes initial slopes in the flexural stress *versus* deflection curves of SHCC prisms steeper and decreases the flexural deflection capacity. These characteristics of fiber-reinforced concrete prisms and conventional SHCC before and after about 300 freeze-thaw cycles are also reported [21,36]. However, flexural behaviors of both engineered cementitious composites (ECCs), *i.e*., a kind of SHCC materials, with fly ash-to-binder (FA/B) ratios of 55% and 70% showed less stiffness and smaller inelastic deformation after 300 freezing and thawing cycles compared to control ECC cured in laboratory air [27]. For lightweight aggregate concrete, it is reported that 200 cycles of freezing and thawing ranging from 20 °C to −20 °C increased the value of effective fracture toughness and fracture energy estimated according to RILEM method [33]. For PVA fiber reinforced ultra-high toughness cementitious composite (UHTCC) with specific compressive strength of 40 MPa, it is noted that the flexural strength decreased slowly with the number of freeze-thaw cycles increasing. The maximum mid-span deflection of UHTCC prism decreased with the number of freeze-thaw cycles increasing [37].

Virgin 2SHCC prisms kept at the standard curing room during 74 days showed more stable flexural behavior than that of 2SHCC prismatic specimens exposed to 300 cycles of freezing and thawing. The flexural strength of 2SHCC increased a little from 6.35 MPa to 6.82 MPa with the number of freezing and thawing cycles increasing from 0 cycle to 300 cycles. However, flexural strength of virgin 2SHCC prism at curing age of 74 days showed about 15% higher than that of freeze-thaw damaged 2SHCC prism with the same curing age.

### 3.4. Direct Tensile Behavior

The applicability of SHCC material is very wide. The sustainable SHCC material developed in this study is applicable for repairing of existing reinforced concrete infrastructures to improve the crack-damage mitigation. However, civil infrastructures may be exposed to external environment such as freeze-thaw cycles. Freezing and thawing exposure could be lead to modification of mechanical properties of sustainable SHCC as a repair material. Therefore, the mechanical properties of 2SHCC must first be evaluated before a practical application of 2SHCC material.

**Figure 7.**
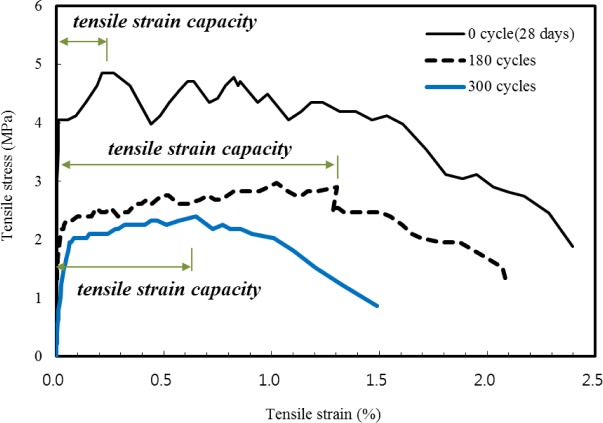
Typical tensile responses of 2SHCC dumbbell-shaped specimens.

Figure 7 shows tensile stress *versus* strain curves of five sustainable SHCC dumbbell-shaped specimens at 0 cycle with a curing age of 28 days, 180 cycles and 300 cycles of freezing and thawing. The stress at the first drop in the direct tensile responses of 2SHCC specimens is defined as the first cracking strength. The maximum stress is also defined as the tensile strength, and the corresponding strain is defined as the tensile strain capacity. Tensile test results are listed in Table 3. As shown in Figure 7 and Table 3, sustainable SHCC mixed in this study exhibits average tensile strength and tensile strain capacity of 4.76 MPa and 0.2% corresponding to yielding strain of steel reinforcement with 400 MPa yield strength, respectively. The tensile strength of 2SHCC experienced 180 and 300 cycles of freezing and thawing decrease 58% and 55% of average tensile strength of virgin 2SHCC, while tensile strain capacity increase 4.7 and 2.8 times of average tensile strain capacity of virgin 2SHCC dumbbell-shaped specimens.

After undergoing 300 freeze-thaw cycles, conventional SHCC tensile specimens with raw components show tensile strength and tensile strain capacity close to those from specimens with the same curing age not subjected to freeze-thaw conditions [2,35]. From these research results, it is seen that freeze-thaw exposure has little effect on the tensile performance of conventional SHCC materials. However, tensile performance of the sustainable SHCC replaced partially with recycled components is slightly different from that of conventional SHCC tensile specimen after repeated freeze-thaw cycles. Due to the old mortar in recycled sand, its water absorption capacity is higher than that of silica sand while the specific gravity of recycled sand is lower than that of silica sand. It is because the relatively high-absorption recycled sand in the 2SHCC resulted in reducing interfacial bond strength between fiber and cement matrix, and cement matrix toughness after freeze-thaw cycling [38,39].

The interfacial transition zone (ITZ) surrounding the fiber in the FRCC is consisted of a higher capillary porosity, the sheet structure of calcium hydroxide and the cluster structure of ettringite crystals. Although the size of the ITZ varies with fiber type and size, most observations suggest that fiber-matrix interface has large porous network structure and relatively weak layer on the order of 40–70 μm thickness that govern FRCC properties [23,28]. When subjected to freezing and thawing cycles, weak porous structure in the fiber-matrix interface deteriorated and is the sensitive area that microcracks occur and propagate wider.

The reduction in tensile strength of 2SHCC dumbbell-shaped specimens subjected to 300 freezing and thawing cycles may be attributed to changes of the fiber-matrix interface. For example, Niu *et al.* [23] and Yun [28] investigated that the interfacial microstructure change of steel fibers and synthetic PVA and PE fibers reinforced cement composites exposed to 300 freezing and thawing cycles, respectively. They observed that after freezing and thawing cycles, the fiber bridging property of FRCCs has improved through a decrease in the chemical bond of the fiber-matrix interface. Consequently, the condition for strain-hardening behavior of FRCC in direct tension is more readily satisfied in the FRCC after freezing and thawing cycles than the same FRCC without freezing and thawing exposure, leading to reduced tensile strength and improved tensile strain capacity.

### 3.5. Bond Behavior between Concrete Substrate and 2SHCC Material

Assuming a uniform tensile stress across the bond plane, the splitting tensile strength from prism splitting tests as shown in Figure 2 was calculated by the following equation [40]:

$$f_{\text{sp}}=\frac{2P}{\pi A}$$

where *f*_sp_ is the splitting tensile strength, P is the maximum applied load, and *A* is the area of bonding plane.

**Figure 8.**
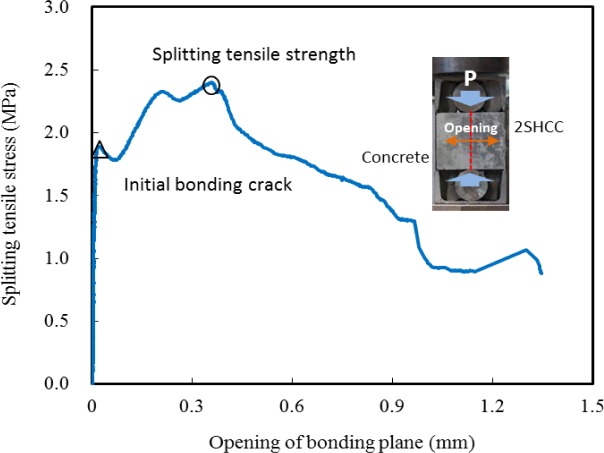
Typical splitting tensile stress *versus* opening displacement of splitting prisms.

Figure 8 presents the typical splitting tensile stress *versus* opening displacement curve of bonding plane in the splitting prism made for evaluating bonding strength between concrete substrate and repair 2SHCC material at 28 days of curing age. An initial crack starts from the loading point to lower support point and for all the specimens, the failure mode was characterized by tension crack along the interface surface. As shown in Figure 8, tensile stress *versus* opening displacement curve of splitting prism is very stiff before an initial bonding crack. Then as splitting load increases, the slope of the curve decreases gradually up to splitting tensile strength. After splitting tensile strength, the bonding crack propagated fast toward lower support and tensile stress decreased.

Table 4 gives the splitting test results, including the mean bond strength, standard deviation, coefficient of variation (COV) and the ratio of the measured bond strengths by different cycles of freezing and thawing to those of virgin specimens. At a curing age of 28 days, the splitting tensile strengths of composite prism specimens averaged 2.59 MPa with a COV of 8.1%. For composite prism specimens stored at the standard curing room during 74 days, the splitting tensile strengths averaged 2.25 MPa with a COV of 4.0%. After 300 freeze-thaw cycles at an age of 74 days, the splitting strengths of prism specimens averaged 1.83 MPa with a COV of 24%. Comparing the splitting tensile strengths of prisms with 90 freeze-thaw cycles with those of prisms with 300 freeze-thaw cycles, there is no difference due to the number of freeze-thaw cycle.

**Figure 9.**
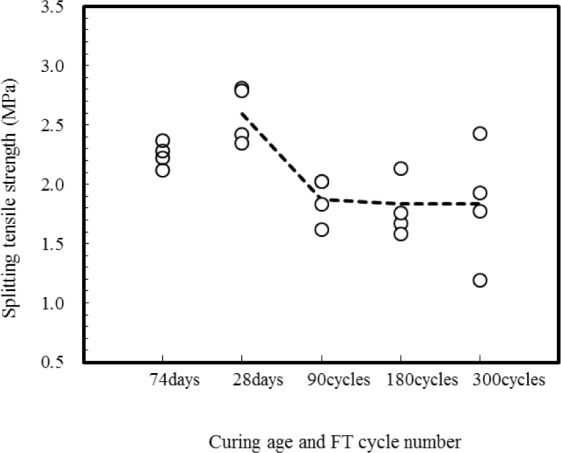
Measured splitting tensile strength of composite prisms.

The effect of freeze-thaw cycling on the bond strength between concrete substrate and repair 2SHCC is shown in Figure 9. The splitting strengths of prism specimens stored at standard curing room decreased from 28 days to 74 days of age. This reduction of splitting strength may have been due to the shrinkage difference between concrete substrate and 2SHCC with rich mixture. The average splitting strength of the freeze-thaw prism specimens was about 30% lower than those of virgin prism specimens with 28 days of curing age. As shown in the splitting test results conducted to evaluate concrete-to-concrete bond strength [29], average splitting strengths of concrete and 2SHCC composite prisms after 300 freeze-thaw cycles indicated 20% lower than those of virgin composite prims at 74 days of age.

## 4. Conclusions

This paper provided the results of compressive, flexural, tensile and splitting tests conducted to investigate the mechanical properties of sustainable strain-hardening cement composite (2SHCC) for civil infrastructures after freeze-thaw actions. Based on the results presented herein, the following conclusions can be drawn:

1. The rapid freeze-thaw cycling of ASTM C 666 Procedure A test up to 300 cycles has little effect on the relative dynamic modulus of elasticity from resonant tests for 2SHCC material used in the study. For 2SHCC prism specimens exposed to 300 freezing and thawing cycles, mass loss remains nearly constant and decreased only less than 1%. The freeze-thaw durability of the 2SHCC was achieved with entraining an adequate size range, number and distribution of air micro-pores into the 2SHCC including fly ash, silica fume and synthetic fibers as components. It can be concluded that the sustainable SHCC material provides equivalent resistance to deterioration in the rapid freezing and thawing environment compared to conventional SHCCs with raw components.
2. The freezing and thawing environment within 300 cycles has little effect on the compressive performance of the sustainable SHCC material, which was in consistency with a previous report on the SHCC materials with raw components. The average compressive strength of 2SHCC material cylinders exposed to 300 freeze-thaw cycles was average 75% of virgin 2SHCC material cylinders stored in the standard curing room during 74 days corresponding to the age of cylindrical specimens experienced 300 freeze-thaw cycles. The significant difference in maturity between 2SHCC cylinders subjected to 300 cycles of freeze-thaw and virgin 2SHCC cylinders would result in the lower compressive strength of freezing and thawing damaged 2SHCC cylinders.
3. The modulus of rupture for 2SHCC material prism does not change appreciably with freeze-thaw cycling in contrast with conventional concrete and steel fiber reinforced concrete, whereas the deflection at the modulus of rupture decreases with freeze-thaw cycling. Like compressive behavior of 2SHCC material, flexural behavior of virgin 2SHCC prism, which is the same curing age of 2SHCC prism exposed to 300 freeze-thaw cycles, is superior to that of freezing and thawing damaged 2SHCC prism.
4. The average direct tensile strength of the 2SHCC dumbbell-shaped specimens with cross-section of 30 mm × 30 mm shows a distinct reduction after freeze-thaw cycling. However, the 2SHCC specimens subjected to 300 freeze-thaw cycles retain more than tensile strain ductility of virgin 2SHCC specimens stored at standard curing room during 28 days. Freezing and thawing cycles deteriorate and weaken the fiber-matrix interface in the 2SHCC. That leads to reduction in the interfacial bond strength that directly influences the strain-hardening behavior of 2SHCC. Consequently tensile strength reduced and tensile strain capacity increase in the 2SHCC after freezing and thawing cycles.
5. For concrete substrate and 2SHCC composite splitting prisms, average splitting tensile strength for a curing age of 28 and 74 days are 2.59 and 2.25 MPa, respectively. With increasing curing age, splitting tensile (bond) strength between the 2SHCC and concrete substrate without freezing and thawing exposure reduced due to the shrinkage difference between substrate and 2SHCC with rich mixture. After exposure to 300 freeze-thaw cycles, average splitting tensile strength of composite prisms shows about 30% reduction compared with that of virgin composite prisms with a curing age of 28 days.

## Figures and Tables

**Table 1.** Mix proportion of sustainable strain-hardening cement composite (2SHCC).

Specified Strength (MPa)	W/B (%)	Unit weight (kg/m^3^)								
		Water	Cement	Fly ash	Silica sand	Recycled sand	MC	SP	Fiber	
									PVA	PET
40	45	444	888	98	355	39.00	0.53	6.91	19.20	5.05

**Table 2.** Compressive strength (*f*_cu_) of 2SHCC material (unit: MPa).

Specimen No.	Virgin 2SHCC		
	28 days	74 days	
1	39.7	45.7	
2	36.6	46.9	
3	37.3	46.2	
Mean *f*_cu_	37.9	46.3	
Standard deviation	1.63	0.60	

**Specimen No.**	**Damaged 2SHCC**		
	**90 cycles**	**180 cycles**	**300 cycles**

1	39.0	36.7	38.8
2	41.3	31.7	36.3
3	38.7	37.8	36.0
Mean *f*_cu_	39.7	35.4	37.0
Standard deviation	1.42	3.25	1.54

**Table 3.** Tensile strength (*f*_t_) of 2SHCC material.

Specimen No.	0 cycle (28-day)			180 cycles			300 cycles		

	First cracking strength (MPa)	Tensile strength (MPa)	Tensile strain capacity (%)	Initial crack strength (MPa)	Tensile strength (MPa)	Tensile strain capacity (%)	Initial crack strength (MPa)	Tensile strength (MPa)	Tensile strain capacity (%)
1	3.18	4.34	0.20	1.01	2.17	0.60	0.79	2.40	0.65
2	3.69	4.85	0.30	1.01	2.54	1.11	0.75	2.47	0.85
3	3.98	4.85	0.25	1.88	2.61	0.57	0.80	2.54	0.35
4	3.11	5.14	0.17	1.89	2.98	1.02	0.79	2.47	0.45
5	3.11	4.63	0.10	2.01	3.48	1.52	1.45	3.12	0.55
Mean *f*_t_	3.41	4.76	0.20	1.56	2.76	0.96	0.92	2.60	0.57
Standard deviation	0.40	0.30	0.08	0.50	0.50	0.39	0.30	0.29	0.19

**Table 4.** Splitting tensile strength(*f*_sp_) of 2SHCC material (unit: MPa).

Specimen No.	Virgin 2SHCC		
	28 days	74 days	
1	2.81	2.37	
2	2.79	2.12	
3	2.41	2.28	
4	2.35	2.22	
Mean *f*_sp(i)_	2.59	2.25	
Standard Deviation	0.21	0.09	
COV, %	8.10	4.00	
*f*_sp(i)_/*f*_sp(28)_	1.00	0.87	

**Specimen No.**	**Damaged 2SHCC**		
	**90 cycles**	**180 cycles**	**300 cycles**

1	1.83	1.67	1.19
2	2.02	2.13	1.93
3	1.62	1.76	1.77
4	2.02	1.58	2.43
Mean *f*_sp(i)_	1.87	1.83	1.83
Standard deviation	0.17	0.29	0.44
COV, %	9.09	18.8	24.0
*f*_sp(i)_/*f*_sp(28)_	0.72	0.71	0.71

## References

[1] Balaguru P.N., Shah S.P. (1992). Fiber-Reinforced Cement Composites.

[2] Zollo R.F. (1997). Fiber-reinforced concrete: An overview after 30 years of development. Cem. Concr. Compos.

[3] Banthia N., Gupta R. (2004). Hybrid fiber reinforced concrete (HyFRC): Fiber synergy in high strength matrices. Mater. Struct.

[4] Choi W.C., Yun H.D., Cho C.G., Feo L. (2014). Attempts to apply high performance fiber-reinforced cement composite (HPFRCC) to infrastructures in South Korea. Compos. Struct.

[5] Li V.C. Advances in Strain-Hardening Cement Based Composites.

[6] Worrell E., Price L., Martin N., Hendricks C., Ozawa M.L. (2001). Carbon dioxide emissions from the global cement industry. Annu. Rev. Energy Environ.

[7] Choi W.C., Yun H.D., Kang J.W., Kim S.W. (2012). Development of recycled strain-hardening cement-based composite (SHCC) for sustainable infrastructures. Compos. Part B Eng.

[8] Li V.C., Lepech M.D., Wang S., Weimann M., Keoleian G.A. Development of Green ECC for Sustainable Infrastructure Systems.

[9] Lepech M.D., Li V.C., Robertson R.E., Keoleian G.A. (2008). Design of green engineered cementitious composites for improved sustainability. ACI Mater. J.

[10] Yang E.H., Yang Y., Li V.C. (2007). Use of high volumes of fly ash to improve ECC mechanical properties and material greenness. ACI Mater. J.

[11] Qian S., Zhou J., de Rooij M.R., Schlangen E., Ye G., van Breugel K. (2009). Self-healing behavior of strain hardening cementitious composites incorporating local waste materials. Cem. Concr. Compos.

[12] Zhou J., Qian S., Beltran M.G.S., Ye G., van Breugel K., Li V.C. (2010). Development of engineered cementitious composites with limestone powder and blast furnace slag. Mater. Struct.

[13] Song S.H., Jang G.S., Cha J.H., Jeon E., Lee G.S., Yun H.D. Mechanical Properties of Green Strain-Hardening Cement Composite with Recycled Fine Aggregates.

[14] Kim S.W., Cha J.H., Kim Y.Y., Yun H.D. (2010). Mechanical properties of strain hardening cement-based composite (SHCC) with recycled materials. J. Korea Concr. Inst.

[15] Altwair N.M., Johari M., Saiyid Hashim S.F. (2012). Flexural performance of green engineered cementitious composites containing high volume of palm oil fuel ash. Constr. Build. Mater.

[16] Huang X., Ranade R., Ni W., Li V.C. (2013). Development of green engineered cementitious composites using iron ore tailings as aggregates. Constr. Build. Mater.

[17] Aldahdooh M.A.A., Muhamad Bunnori N., Megat Johari M.A. (2014). Influence of palm oil fuel ash on ultimate flexural and uniaxial tensile strength of green ultra-high performance fiber reinforced cementitious composites. Mater. Des.

[18] Yun H.D., Rokugo K., Park W.S. Mechanical Properties of Sustainable Strain-Hardening Cement-Based Composites after Exposure to Rapid Freeze-Thaw Cycles.

[19] Philleo R.E. (1986). Freezing and thawing resistance of high-strength concrete. NCHRP Synthesis of Highway Practice.

[20] Janssen D.J., Snyder M.B. (1994). Resistance of Concrete to Freezing and Thawing. Strategic Highway Research Program.

[21] Yun H.D., Rokugo K. (2012). Freeze-thaw influence on the flexural properties of ductile fiber-reinforced cementitious composites (DFRCCs) for durable infrastructures. Cold Reg. Sci. Technol.

[22] Richardson A.E., Coventry K.A., Wilkinson S. (2012). Freeze/thaw durability of concrete with synthetic fibre additions. Cold Reg. Sci. Technol.

[23] Niu D., Jiang L., Bai M., Miao Y. (2013). Study of the performance of steel fiber reinforced concrete to water and salt freezing condition. Mater. Des.

[24] Jin S., Zhang J., Huang B. (2013). Fractal analysis of effect of air void on freeze-thaw resistance of concrete. Constr. Build. Mater.

[25] Lepech M.D., Li V.C. (2006). Long term durability performance of engineered cementitious composites. Restor. Build. Monum.

[26] Ahmed S.F.U., Mihashi H. (2007). A review on durability properties of strain hardening fibre reinforced cementitious composites (SHFRCC). Cem. Concr. Compos.

[27] Şahmaran M., Özbay E., Yücel H.E., Lachemi M., Li V.C. (2012). Frost resistance and microstructure of engineered cementitious composites: Influence of fly ash and micro poly-vinyl-alcohol fiber. Cem. Concr. Compos.

[28] Yun H.D. (2013). Effect of accelerated freeze-thaw cycling on mechanical properties of hybrid PVA and PE fiber-reinforced strain-hardening cement-based composites (SHCCs). Compos. Part B Eng.

[29] Geissert D.G., Li S., Frantz G.C., Stephens J.E. (1999). Splitting prism test method to evaluate concrete-to-concrete bond strength. ACI Mater. J.

[30] Japan Society of Civil Engineers (2008). Recommendations for Design and Construction of High Performance Fiber Reinforced Cement Composites with Multiple Fine Cracks (HPFRCC).

[31] Pigeon M., Pleau R., Azzabi M., Banthia N. (1996). Durability of microfiber-reinforced mortars. Cem. Concr. Res.

[32] Won J.P., Jang C.I., Lee S.W., Lee S.J., Kim H.Y. (2010). Long-term performance of recycled PET fibre-reinforced cement composites. Constr. Build. Mater.

[33] Kucharczyková B., Keršner Z., Pospíchal O., Misák P., Vymazal T. (2010). Influence of freeze-thaw cycles on fracture parameters values of lightweight concrete. Procedia Eng.

[34] Shang H., Song Y., Ou J. (2009). Behavior of air-entrained concrete after freeze-thaw cycles. Acta Mech. Solida Sin.

[35] Li V.C., Fischer G., Kim Y.Y., Lepech M.D., Qian S., Weimann M., Wang S. (2003). Durable Link Slabs for Jointless Bridge Decks Based on Strain-Hardening Cementitious Composites.

[36] Balaguru P.N., Ramakrishnan V. (1986). Freeze-thaw durability of fiber reinforced concrete. ACI J.

[37] Xu S., Cai X. (2010). Mechanics behavior of ultra high toughness cementitious composites after freezing and thawing. J. Wuhan Univ. Technol. Mater. Sci. Ed.

[38] Wang S., Li V.C. (2006). High-early-strength engineered cementitious composites. ACI Mater. J.

[39] Salem R.M., Burdette E.G., Jackson N.M. (2003). Resistance to freezing and thawing of recycled aggregate concrete. ACI Mater. J.

[40] Nilsson S. (1961). The tensile stress of concrete determined by splitting tests on cubes. RILEM Bull. Paris.

